# Ultrafast Excited-State Dynamics of Dithienyltetrazine-Based Donor–Acceptor Copolymers

**DOI:** 10.1021/acs.jpcb.6c01609

**Published:** 2026-05-25

**Authors:** Erico M. Braun, Samylla Boazegevski, Jeferson F. de Deus, Cristiano Zanlorenzi, Sandra M. Cassemiro, Leni C. Akcelrud, Samim Sardar, Franco V. A. Camargo, Giulio Cerullo, Giovanni Bressan, Anam Fatima, Stephen R. Meech, Ismael A. Heisler

**Affiliations:** 1 Instituto de Física, 28124 Universidade Federal do Rio Grande do Sul - UFRGS, Avenida Bento Gonçalves, 9500, Porto Alegre 90040-060, Brazil; 2 Departamento de Física, 74354 Universidade Tecnológica Federal do Paraná − UTFPR, Av. Sete de Setembro, 3165, Curitiba 80230-901, Brazil; 3 Departamento de Química, 28122 Universidade Federal do Paraná - UFPR, 81531-990 Curitiba, Brazil; 4 Department of Chemistry, 28673 National Institute of Technology Warangal, Warangal, Telangana 506004, India; 5 Istituto di Fotonica e Nanotecnologie, 9327 Consiglio Nazionale delle Ricerche, Piazza L. da Vinci 32, 20133 Milano, Italy; 6 Dipartimento di Fisica, 18981 Politecnico di Milano, Piazza L. da Vinci 32, 20133 Milano, Italy; 7 School of Chemistry, Norwich Research Park, 6106 University of East Anglia,Norwich NR4 7TJ, U.K.

## Abstract

Tetrazine-based donor–acceptor (D–A)-conjugated polymers exhibit strong optical absorption and tunable electronic structures, yet they often suffer from weak photoluminescence and limited optoelectronic performance. The microscopic origins of this fluorescence quenching, particularly on ultrafast time scales, remain insufficiently understood. Here, we investigate the excited-state dynamics of dithienyltetrazine (**TTz**) and two D–A copolymers, poly­(carbazole–TTz) (**PCTTz**) and poly­(indolocarbazole–TTz) (**PICTTz**), using a combination of steady-state spectroscopy, time-resolved fluorescence upconversion, time-correlated single photon counting, femtosecond transient absorption spectroscopy, and complementary density functional theory calculations. We show that photoexcitation of the copolymers initially populates a strongly allowed intramolecular charge-transfer (ICT) state, which undergoes ultrafast (<100 fs) internal conversion to a weakly emissive tetrazine-localized singlet n−π* state, consistent with partial population of long-lived states likely involving the triplet manifold. Steady-state and time-resolved measurements reveal pronounced conformational heterogeneity, with a minor subpopulation of highly twisted polymer segments suppressing ICT formation and enabling brighter, TTz-like emission at higher excitation energies. Comparison between **PCTTz** and **PICTTz** further demonstrates that subtle variations in donor structure critically influence the lifetime and deactivation pathways of the dark singlet excited state, leading to either nanosecond singlet lifetime or rapid internal conversion. These findings establish a unified mechanistic framework for fluorescence quenching in tetrazine-based D–A polymers and provide clear design guidelines for mitigating ultrafast internal conversion in future organic optoelectronic materials.

## Graphical abstract

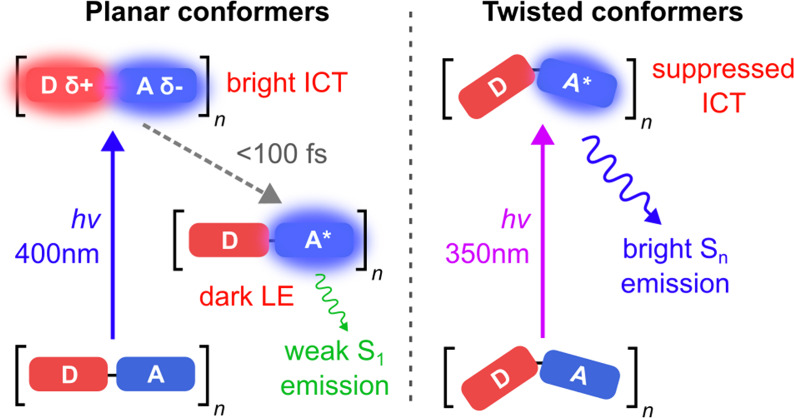

## 1 Introduction

Conjugated donor–acceptor (D–A) polymers have become a cornerstone of modern organic optoelectronics, underpinning technologies such as organic photovoltaics (OPVs), photodetectors, light-emitting diodes, and photocatalytic systems.^1–3^ Their success arises from the ability to tailor electronic structure through molecular design, enabling precise control over optical gaps, absorption bandwidths, frontier orbital alignment, and excited-state character.^4–6^ In D–A architectures, photoexcitation typically generates an intramolecular charge-transfer (ICT) state, and the formation, relaxation, and deactivation pathways of this state ultimately govern key material properties including radiative efficiency, exciton diffusion, and charge separation.^7–10^ A detailed understanding of ICT-state dynamics, particularly on ultrafast time scales, is therefore essential for establishing structure–property relationships and guiding rational materials optimization.^11–13^

A widely used strategy for engineering low-bandgap D–A polymers is the incorporation of strong electron-withdrawing acceptor units.^14–16^ Among these, *s*-tetrazine derivatives occupy a distinctive position owing to their high electron affinity, compact heteroaromatic framework, and synthetic versatility.^17,18^ Tetrazine-based copolymers frequently exhibit broad and intense absorption bands extending across the visible spectrum, attributes that make them appealing candidates for OPV active layers. Despite these favorable absorption characteristics, however, tetrazine-containing polymers often display weak photoluminescence and modest device efficiencies.^18,19^ This apparent contradiction suggests the presence of highly efficient excited-state deactivation pathways that compete with radiative decay and limit the functional performance of these materials.

The photophysical origin of this behavior can be traced back to the electronic structure of tetrazine units.^17,18,20^ Studies on tetrazine molecules have established that a n−π* S_1_ state lies energetically close to the optically bright π–π transitions, facilitating rapid internal conversion and realizing low fluorescence quantum yields.^18,20^ Besides rapid population of a dark S_1_, other recent studies have also analyzed the role of intersystem crossing (ISC) in tetrazine-based fluorophores and OPVs, as its n−π* S_1_ has a favorable ISC condition by the El-Sayed rules.^21–23^ When tetrazines are incorporated into extended conjugated backbones, this intrinsic propensity for nonradiative decay is further modulated by conformational disorder along the polymer chain. Thermal fluctuations and torsional degrees of freedom generate a heterogeneous ensemble of chromophores with varying donor–acceptor coupling strengths, each sampling a distinct local excited-state landscape.^24–26^ As a consequence, the photophysical response of tetrazine-based polymers reflects an average over multiple emissive and nonemissive pathways, complicating the identification of the microscopic mechanisms responsible for fluorescence quenching.

Steady-state spectroscopic measurements alone are often insufficient to disentangle this complexity.^25,27^ In many tetrazine-based copolymers, strong absorption bands coexist with weak and excitation-dependent fluorescence, implying that the initially populated bright excited state is rapidly depopulated prior to emission. Whether this quenching proceeds through direct internal conversion to the ground state or via population of a low-lying triplet state remains an open question.^28^ Moreover, the extent to which backbone conformation controls access to these pathways—either by promoting efficient ICT formation or by electronically decoupling donor and acceptor units—has yet to be fully clarified.^29^ In previous work^30^ on fluorene-based conjugated polymers, ultrafast spectroscopy has shown that excited-state dynamics are primarily governed by exciton relaxation and localization within a distribution of chromophores. In contrast, tetrazine-based donor–acceptor systems introduce low-lying n−π* states that fundamentally alter the relaxation pathways. As a result, the excited-state evolution is no longer dominated by structural energy funneling but instead by ultrafast population of dark states, providing an efficient nonradiative decay channel that directly impacts photoluminescence efficiency.

Recent studies on tetrazine-based OPV materials have underscored the detrimental impact of ultrafast excited-state deactivation on device performance.^31,32^ Rapid trapping of photoexcited population into nonradiative states suppresses down-chain energy transfer and limits the formation of long-lived charge-separated states, ultimately reducing photocurrent generation. Despite growing recognition of this issue, direct experimental insight into the earliest stages of excited-state evolution in tetrazine-based D–A polymers remains scarce, largely due to the need for spectroscopic techniques capable of resolving subpicosecond dynamics while selectively probing emissive and nonemissive states.^18^

In this work, we present a comprehensive investigation of the excited-state dynamics of dithienyltetrazine (**TTz**) and two structurally related D–A copolymers, poly­(carbazole–TTz) (**PCTTz**) and poly­(indolocarbazole–TTz) (**PICTTz**). These systems provide an ideal platform for isolating the roles of molecular structure, donor–acceptor coupling, and conformational disorder in determining excited-state relaxation pathways. By combining steady-state absorption and emission spectroscopy with time-resolved fluorescence upconversion, time-correlated single photon counting, femtosecond transient absorption spectroscopy, and complementary density functional theory calculations, we track the evolution of photoexcited states from femtoseconds to nanoseconds.

Our results provide a unified mechanistic picture in which photoexcitation of the copolymers initially populates a strongly allowed ICT state that undergoes ultrafast (<100 fs) internal conversion (IC) to a tetrazine-localized n−π* locally excited (LE) S_1_ state with weak emissivity. This rapid funneling into a dark state provides a direct explanation for the low fluorescence quantum yields observed despite strong optical absorption. Crucially, we further identify an ISC pathway due to spin–orbit coupling of the n−π* S_1_ to energetically close π–π* T_*n*_ states. Besides tracking the evolution of the dark S_1_, we also identified a minor subpopulation of highly twisted polymer conformers in which donor–acceptor conjugation is disrupted, suppressing ICT formation but enabling brighter, TTz-like emission from excited states above S_1_. Comparison between **PCTTz** and **PICTTz** demonstrates that subtle variations in donor structure critically influence the lifetime and decay pathways of the dark LE S_1_ state, leading either to rapid ground-state recovery through S_1_→S_0_ IC or to long-lived triplet excited states.

## 2 Methods

### 2.1 Experimental Section

Details for each experimental technique are found in the Supporting Information. Samples were synthesized following ref 31 and were dissolved in spectroscopy-grade chloroform (Sigma-Aldrich) for all measurements. Solutions were prepared in 10 mm path length cuvettes with OD 0.5 for UV–vis and <0.1 for fluorescence spectroscopies. Time-correlated single photon counting (TCSPC) was conducted with the same solutions prepared for fluorescence using either 365 or 485 nm excitation and detected the emission at 535 nm, with the instrument response function (IRF) width determined to be 900 and 470 ps (fwhm) for each excitation source, respectively. Femtosecond transient absorption (TA) spectroscopy was performed using a well-established setup,^27,33^ with pump wavelength being either 350, 400, or 490 nm (100 fs IRF width in all cases) and polarization set to the magic angle relative to the white light probe obtained by supercontinuum generation in a CaF_2_ plate. Time-resolved fluorescence upconversion (TRFU) used a setup previously described in the literature,^34^ with a fixed 400 nm excitation (82 fs IRF width) and detection at 500–510 nm. Samples for TA and TRFU measurements were prepared in static 1 mm path length cuvettes, with OD values in the range 0.3–0.5 at the excitation wavelength.

### 2.2 Computational Method

Density functional theory (DFT) calculations were performed at the ωB97X-D3/def2-TZVP level of theory as implemented in ORCA 6.1 ^35,36^ to optimize ground-state geometries of the three samples. Geometries were optimized using a conductor-like polarizable continuum model (CPCM) of chloroform until no imaginary frequencies were found. To decrease computational effort, alkyl side chains were replaced by methyl groups and copolymers consisted of a single repeat unit (one donor–acceptor pair). Time-dependent DFT (TDDFT) was employed at the same level of theory to calculate vertical transition energies and oscillator strengths from the optimized ground-state geometries, as well for S_1_ geometry optimization and spin–orbit couplings at this geometry. Visualization of the molecular orbitals was carried out using Avogadro.^37^

## 3 Results and Discussion

A general scheme of the molecular structures of the **TTz** monomer and the donor–acceptor copolymers **PCTTz** and **PICTTz** is presented in Figure 1. Normalized steady-state absorption and emission spectra of each sample in chloroform are shown in Figure 2 with the emission corresponding to excitation at the absorption maximum of each sample. The use of low concentrations in a suitable solvent ensures that the polymer chains are fully solvated so that intermolecular interactions among neighboring chains are not significant. The **TTz** absorption spectrum (Figure 2a) shows a strongly allowed transition at 377 nm and two weaker transitions at 445 and 535 nm.

**Figure 1:**
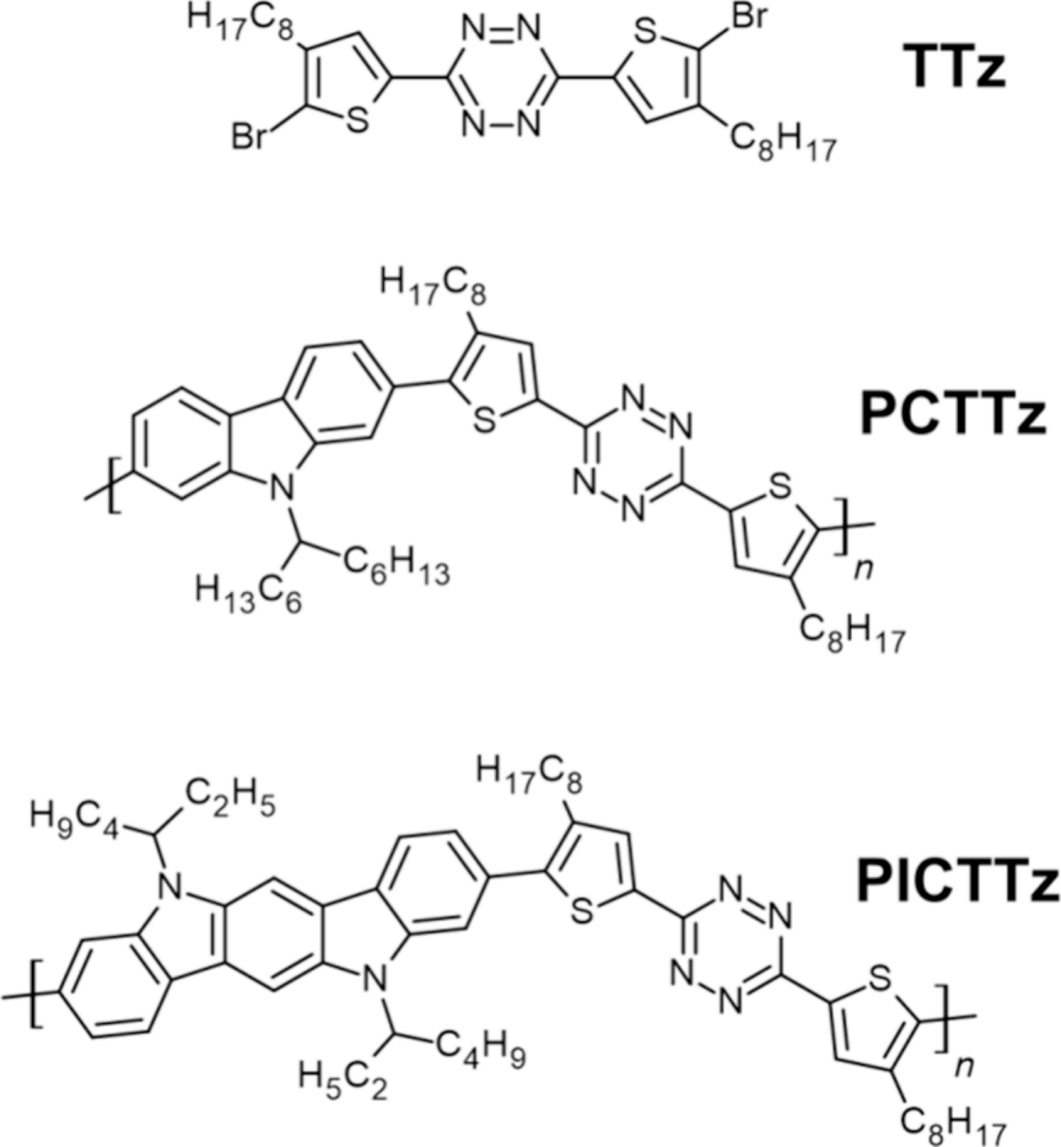
Molecular structures of dithienyltetrazine (**TTz**) and its derivatives copolymers **PCTTz** and **PICTTz**.

**Figure 2:**
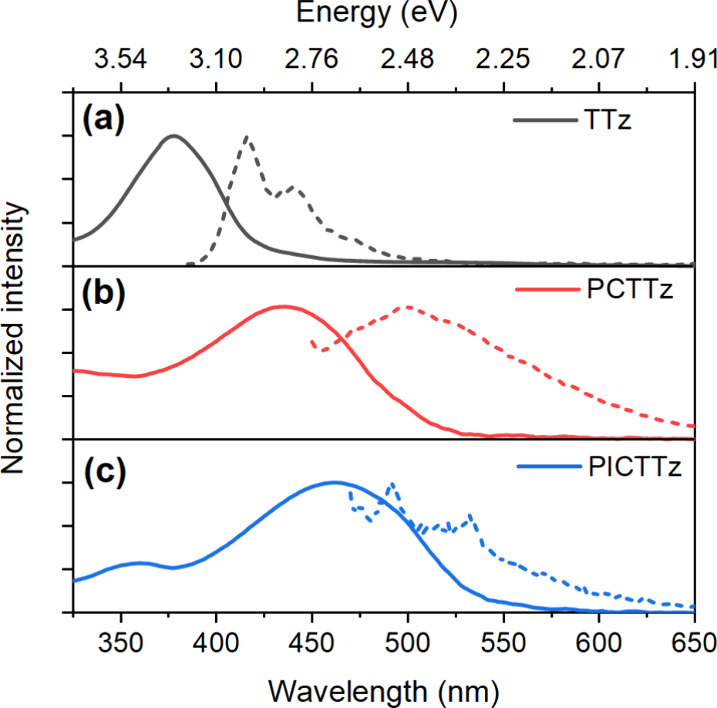
Normalized steady-state absorption (solid) and emission spectra (dashed) under excitation at the absorption maximum for (a) **TTz** (black), (b) **PCTTz** (red), and (c) **PICTTz** (blue).

These transitions in **TTz** were previously attributed as S_0_→S_3_ at 377 nm (3.29 eV) involving π–π* orbitals delocalized over the three constituent rings, S_0_→S_2_ at 445 nm (2.79 eV) and S_0_→S_1_ at 535 nm (2.32 eV) with considerably weaker oscillator strengths, as they involve a π–π* and n−π* transition, respectively.^18,20^ Absorption spectra of the copolymers **PCTTz** and **PICTTz** (Figure 2b,c) show a decrease of the optical gap relative to **TTz** due to their donor–acceptor designs, since this design promotes an ICT state, which is mostly affected by the donor HOMO and acceptor LUMO energies.^4,38^ The lowest absorption band for **PCTTz** is located at 436 nm (2.84 eV), while **PICTTz** is at 461 nm (2.69 eV), with the larger redshift attributable to the higher energy of the HOMO of the indolocarbazole donor (−5.0 eV vs carbazole −5.7 eV).^39^ The overall line shape of the copolymers’ absorption spectra is also similar to one another, both displaying inhomogeneously broadened optical transitions, a typical feature of polymeric chains, where the random distribution of conformations results in strong transition broadening.^26,40^

In terms of emission, excitation at the absorption peak in **TTz** (S_0_→S_3_) leads to a vibronic progression profile, with peaks at 416, 440, and 469 nm, corresponding to a spacing of ∼1350 cm^–1^. Because the lowest singlet state of **TTz** has n−π* character and very low oscillator strength, fluorescence can originate from higher π–π* states before internal conversion to S_1_, leading to apparent S_*n*_ emission.^20,22^ The copolymers, however, exhibit a featureless fluorescence spectra under excitation at their respective absorption maxima. Their emission also displays a pronounced dependence on the excitation wavelength (λ_exc_), as shown in Figure 3a for **PCTTz** (fluorescence data and excitation dependence for **PICTTz** were previously reported by Cassemiro et.al.^31^). When excited at its absorption maximum (436 nm), **PCTTz** exhibits only a weak emission peaking at ∼500 nm. However, as λ_exc_ is shifted to shorter wavelengths (410, 400, 375, and 350 nm), the emission peak moves concomitantly to shorter wavelengths and its intensity increases dramatically, reaching nearly an order of magnitude enhancement compared to excitation at 436 nm (Figure 3a) despite the ∼50% decrease in absorbance. Scanning the excitation and monitoring **PCTTz** emission at 520 nm (Figure 3b) reveals a distinct excitation band centered around 363 nm, well above the absorption maximum.

**Figure 3:**
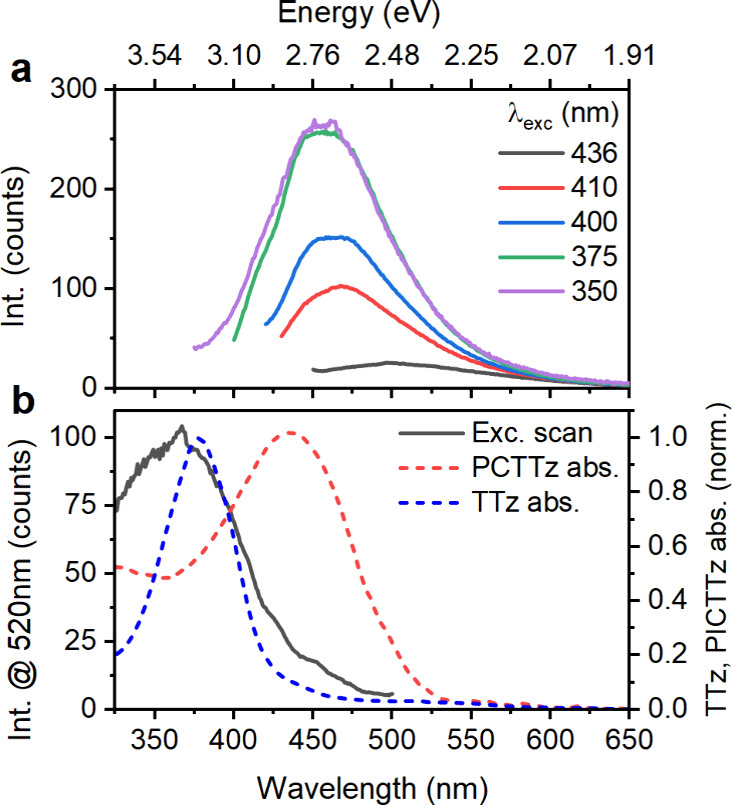
(a) Excitation wavelength λ_exc_ dependence of **PCTTz** emission. (b) Excitation scan at 520 nm emission of **PCTTz** (solid) compared to its absorption and **TTz** absorption (dashed, a.u.).

To compare the brightness of the excitation-dependent emission of the copolymers, we performed relative fluorescence quantum yield (FQY, Φ_f_) measurements using the dye coumarin 7 (Φ_f_ = 0.82 in methanol^41^) as a reference. The calculated FQY values from the steady-state fluorescence are presented in Table 1 for excitation wavelengths of 380, 400, 430, and 460 nm. Both copolymers exhibited an increase of their FQY when the excitation moved to shorter wavelengths, with **PCTTz** FQY increasing from 1 × 10^–3^ (460 nm excitation) to 0.07 (380 nm) and **PICTTz** FQY from 10^–5^ (460 nm) to 5 × 10^–3^ (380 nm). For the same excitation wavelengths, **PCTTz** showed a consistently higher FQY value than **PICTTz** by at least an order of magnitude. For comparison, a similar dithienyltetrazine has a reported FQY of ∼6 × 10^–4^ in cyclohexane,^20^ while other *s*-tetrazine-based fluorophores exhibit typical FQY values in a range of 10^–3^ to 0.04, only going as high as ∼0.3 with certain chlorine substituents.^23,42^

**Table 1:** Excitation Dependence of the Fluorescence Quantum Yield (FQY) in **PCTTz** and **PICTTz** ^a^

excitation (nm)	**PCTTz** Φ_f_	**PICTTz** Φ_f_
380	0.07	5 × 10^–3^
400	0.01	1 × 10^–3^
430	2 × 10^–3^	10^–4^
460	1 × 10^–3^	10^–5^

a FQYs were determined using coumarin 7 (Φ_f_ = 0.82) as a reference standard (ref 41). Excitation wavelengths were chosen to maximize overlap between the absorption bands of the two copolymers and the standard. The reported FQY value for **TTz** (∼6 × 10^–4^ in cyclohexane) is taken from ref 20.

The disparity between the absorption and excitation spectra in both copolymers and increase of the FQY at shorter excitation wavelengths indicate the presence of a heterogeneous structure distribution in solution. While the majority of the structures is weakly or nonemissive and responsible for the lowest-energy absorption band, the emission of both copolymers is dominated by a subgroup of structures that contributes mostly to the higher-lying absorption band at 350–380 nm (Figure 3b). The nature of these emissive subpopulations can be inferred by comparison with the steady-state absorption and fluorescence spectra of the **TTz** monomer (Figure 2a), which displays its S_0_→S_3_ transition in the same region as to where the copolymer subpopulations absorb (Figure 3b). Tuning the excitation wavelength specifically to this subpopulation in the copolymers, their emission mostly coincides with **TTz**’s S_*n*_ emission region (400–500 nm), suggesting the existence of conformers where π-conjugation is largely confined to the acceptor unit, enabling emission from S_*n*_ (*n* > 1) in the copolymers similar to the one found in the **TTz** monomer.^22^ This scenario is similar to previous reports for PM6 polymers,^43^ where highly twisted conformers break π-conjugation and enable bright emission from a subpopulation at shorter wavelengths.

The character of the transitions reported above was further investigated using DFT and TDDFT at the ωB97X-D3/def2-TZVP level of theory as implemented in ORCA 6.1.^36^ Solvent influence was accounted for using CPCM of chloroform. The optimized DFT ground-state geometries are found in Figure 4a, which shows **TTz** adopting a planar configuration in its electronic ground state. **PCTTz** and **PICTTz** on the other hand show a dihedral angle of 43.4° between their mostly planar donor and acceptor moieties. Relevant difference densities, oscillator strengths, and vertical transition energies calculated via TDDFT for the first three excited states of all samples are summarized in Figure 4b,c (all 10 first singlet excitations are found in Tables S1–S3 and comparison with absorption spectra in Figure S11). The calculations agree well with the steady-state absorption spectrum of **TTz**, showing three major transitions: first a weak (*f* = 0.0096) n−π* transition from the HOMO–2 to LUMO at 2.609 eV (475 nm) involving orbitals localized at the tetrazine ring, a further weaker (*f* = 0.0001) HOMO–2→LUMO+1 at 3.737 eV (332 nm) from an n orbital to a π* orbital delocalized over the entire molecule, and finally, a strong (*f* = 1.4212) HOMO→LUMO+1 π–π* transition at 3.920 eV (316 nm) involving orbitals delocalized over the entire molecule. We note that oscillator strengths larger than unity can arise in multielectron systems due to redistribution of transition strength, as described by the Thomas–Reiche–Kuhn sum rule. We also highlight that when the tetrazine n orbital is located below the HOMO, negligible emission from S_1_ has been reported,^18^ a trend which is also observed in this work. TDDFT calculations of the copolymers follow a similar trend, with the S_0_→S_1_ transition at 2.595 eV (478 nm) exhibiting a very similar character to the one calculated for **TTz** in both copolymers. This transition involves the n−π* orbitals localized on the *s*-tetrazine heterocycle (HOMO–4→LUMO in **PCTTz** and HOMO–5→LUMO in **PICTTz**) and thus labeled as locally excited (LE). Two higher transitions appear very close in energy, with S_0_→S_2_ and S_0_→S_3_ at 3.753 and 3.798 eV (330 and 326 nm) for **PCTTz** and 3.736 and 3.754 eV (332 and 330 nm) for **PICTTz**, respectively. Like in **TTz**, the transitions at ∼3.75 eV are from an *s*-tetrazine n orbital to a π* orbital of the dithienyltetrazine (HOMO–4→LUMO+1 in **PCTTz** and HOMO–5→LUMO+1 in **PICTTz**), with very similar transition energies and small oscillator strengths (Figure 4b). In contrast, the calculated S_0_→S_3_ in **PCTTz** and S_0_→S_2_ in **PICTTz** have significant ICT character, involving a transition from a donor π orbital to an acceptor π* orbital (HOMO→LUMO+1 in **PCTTz** and HOMO–1→LUMO+1 in **PICTTz**, Figure 4b,c), carrying significant oscillator strength (*f* > 1.9). Considering the high oscillator strength of the ICT transitions in **PCTTz** and **PICTTz**, they are assigned as the strongly allowed absorption bands of each sample at 436 and 461 nm, respectively. The calculated vertical transition energy to the bright ICT state appears higher in energy (326 and 332 nm for **PCTTz** and **PICTTz**, respectively) when compared to the measured values, which may be due to the calculation only considering one donor–acceptor pair. This does not hinder this assignment as no other strongly allowed transition was found in the calculations nearby those spectral regions (Tables S1–S3).

**Figure 4:**
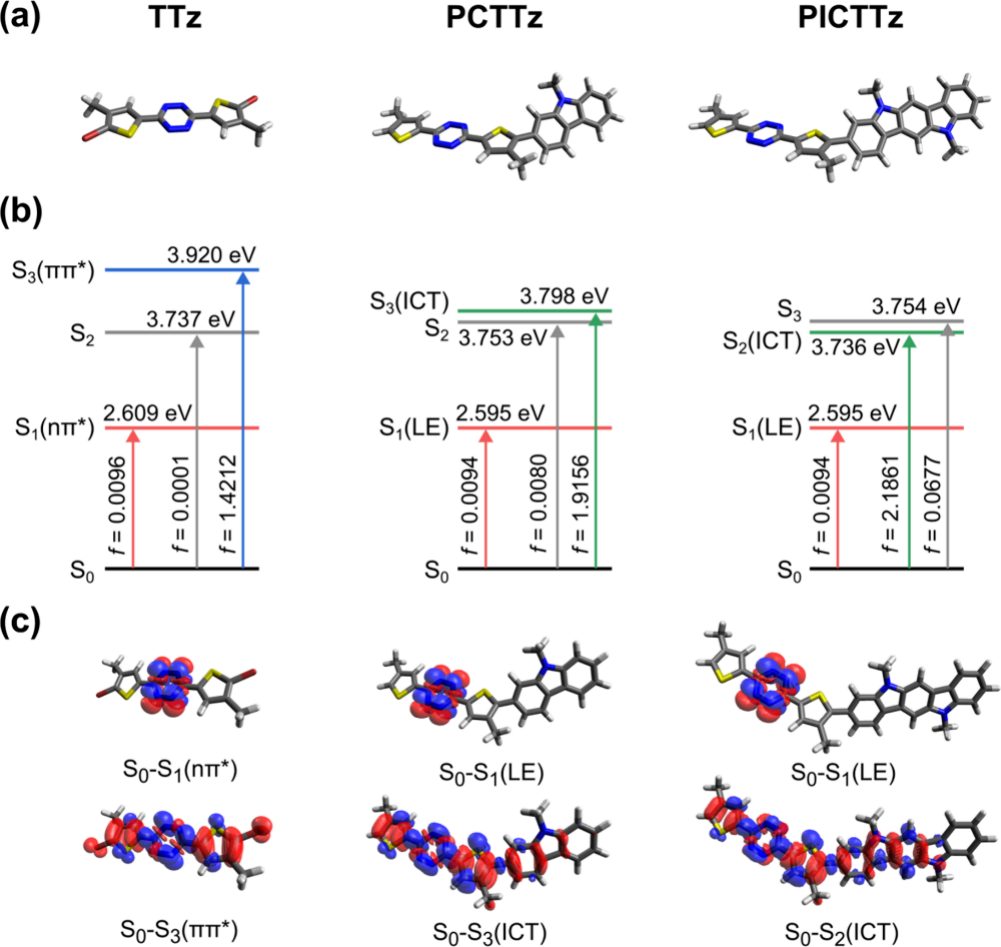
(a) Optimized ground-state geometries for **TTz**, **PCTTz**, and **PICTTz** at the ωB97X-D3/def2-TZVP level of theory. (b) Calculated excitation energies and oscillator strengths for the first three vertical transitions at the same level of theory. (c) Difference densities (blue: electron, red: hole) for the first and most dipole-allowed excited states.

Excitation energies and oscillator strengths were also performed at the S_1_ optimized geometry at the same level of theory (Figure S12). It was observed that S_1_–S_0_ energy gap decreases to about 2.12 eV (585 nm) in all samples, with a concomitant decrease of the S_1_→S_0_ oscillator strength to *f* ∼ 0.006. Triplet energies and spin–orbit couplings (SOCs) from S_1_ were also computed at this geometry. With S_1_ exhibiting n−π* character, SOCs are enhanced to π–π* triplet states, with calculated couplings ranging from 1 to 15 cm^–1^ between T_2_ and T_4_ (Figure S12). The calculated S_1_–T_2_ energy gap is similar in all samples, being ∼0.2 eV in **TTz**, ∼0.3 eV in **PCTTz**, and ∼0.2 eV in **PICTTz**.

Next, we proceed to report a detailed investigation of the excited-state dynamics for the dithienyltetrazine-based structures, starting with time-resolved fluorescence upconversion (TRFU). This is a key method for elucidating the initial excited-state events governing the emissive properties of the copolymers. Its high temporal resolution (<80 fs) and sensitivity to emissive states only make it the ideal technique to probe the decay of strongly emissive species. The resulting fluorescence decays, presented in Figure 5, show an exceptionally rapid decay channel for both copolymers. Fitting the traces to a parallel exponential decay model reveals that the majority of the initial amplitude decays through a component with time constants of 51 fs (93% amplitude) for **PCTTz** and 57 fs (96%) for **PICTTz**. Subsequently, a minor slower process with time constants of 1.2 ps (5%, **PCTTz**) and 1.5 ps (4%, **PICTTz**) follows the ultrafast relaxation. While the emission from **PICTTz** is fully quenched after the minor process, **PCTTz** exhibits a persistent emission lasting beyond the 20 ps of delay that was measured and that was accounted for in the fit as a constant offset. Such an ultrafast time scale and large amplitude of the initial decay point to an essentially barrierless fluorescence quenching process on the excited-state potential energy surface in both copolymers, which, based on our calculations, can be attributed to the conversion of the initially populated (bright) ICT state to the weakly emissive (dark) S_1_(LE) state through an ultrafast IC.

**Figure 5:**
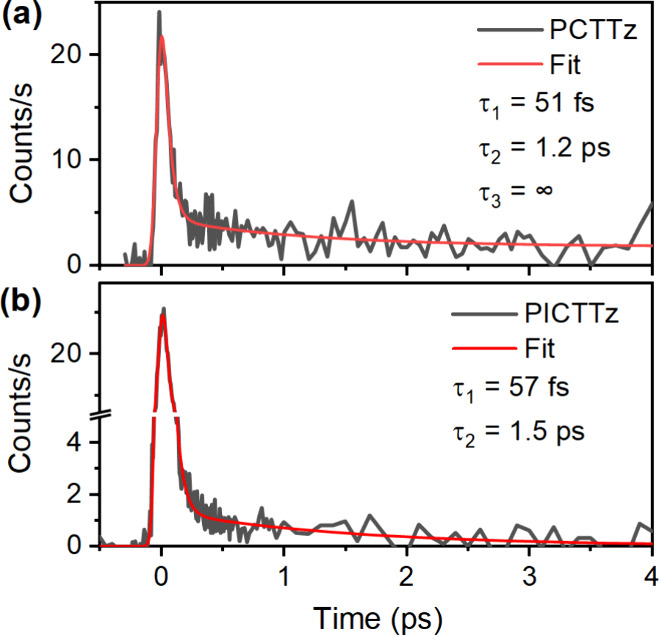
TRFU traces of (a) **PCTTz** and (b) **PICTTz** in counts per second above baseline. The detection wavelengths are 510 and 500 nm, respectively. IRF width for all TRFU measurements is 82 fs.

To quantify the long-lived emission of **PCTTz**, we used time-correlated single photon counting (TCSPC). As **PCTTz** exhibits excitation wavelength-dependent emission, TCSPC was measured using two excitation wavelengths of 365 and 485 nm, targeting the strongly and weakly emissive populations, respectively (Figure S1). Both excitation wavelengths yielded a biexponential emission decay, with lifetimes of 0.96 ± 0.02 and 3.1 ± 0.3 ns (with 92% and 8% amplitude, respectively) when excited at 365 nm and 1.42 ± 0.02 and 3.79 ± 0.05 ns (51% and 49%, respectively) when excited at 485 nm. This difference in **PCTTz**’s nanosecond-scale emission serves as direct kinetic evidence for conformational heterogeneity within the solvated polymer chains. While the weakly emissive population promotes a slower biexponential decay with practically equal contribution from the two decay channels, the brighter subpopulation, with its uncoupled D–A units, recovers faster and preferentially through the 0.96 ns channel. While a value of 0.96 ns is likely IRF-limited, it suggests a suppressed radiationless decay of the bright subpopulation due to the large S_*n*_–S_1_ energy gap of LE states in the TTz subunit.^22^

To further investigate the dynamics after the decay of the strongly emissive states, we performed femtosecond transient absorption (TA) spectroscopy in solution to investigate if any weakly emissive species, either singlets or triplets, are present. The pump fluence was maintained in the 5–10 μJ/cm^2^ range, ensuring both a high signal-to-noise ratio and operation within the linear regime to avoid multiexciton generation and other higher order effects (the fluence dependence for the copolymers is found in Figures S2 and S3). In these spectra, presented as a relative transmission change (Δ*T*/*T*), positive features are due to ground-state bleach (GSB) and stimulated emission (SE), while negative features correspond to excited-state absorption (ESA).

Figure 6 shows the TA data for all the samples following photoexcitation by a 350 nm pump for **TTz** and 400 nm for **PCTTz** and **PICTTz**. The early time spectra of the three samples are characterized by a strong positive signal flanked by two distinct negative ESA bands. In **TTz** (Figure 6a), the positive signal covers the 340–410 nm region, assigned as GSB and SE. For **PCTTz** (Figure 6b), the positive contribution is found in the region from 370 to 500 nm, while for **PICTTz** (Figure 6c), the positive feature extends from 420 to 535 nm, both coinciding well with their respective steady-state absorption profiles and thus primarily assigned to GSB. While some SE may contribute to this positive feature, the small emissivity of the excited state precludes a definitive assignment. Notably, the GSB features in the copolymers are narrower than the steady-state absorption, a common characteristic in polymers attributed to inhomogeneous broadening, where the ultrafast pump selectively excites a subset of ground-state conformations.^25,30^ Regarding the negative features, the TA spectrum of **TTz** starts with a weak ESA band below 340 nm and a stronger one above 410 nm. **PCTTz** also displays an ESA band below 370 nm and a notably stronger, broad ESA band above 500 nm. Conversely, the strength of the ESA bands is the opposite in **PICTTz**, which exhibits a prominent ESA band covering the 340–420 nm region, whereas its second ESA band above 535 nm is weaker, broad, and featureless.

**Figure 6:**
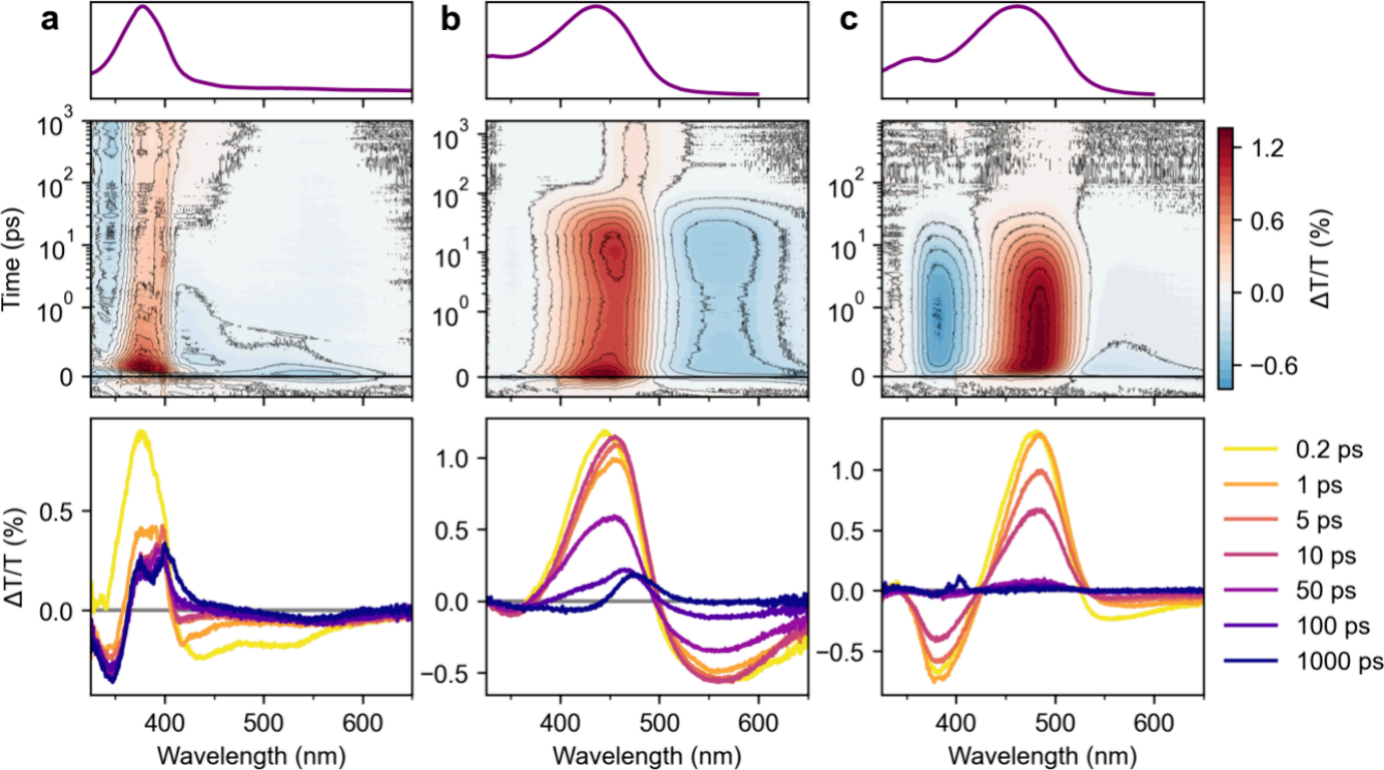
Steady-state absorption spectra (top row), TA maps (middle), and TA spectra (bottom) at selected delay times between 0.2 and 1000 ps for (column a) **TTz** following 350 nm excitation, (column b) **PCTTz**, and (column c) **PICTTz** following 400 nm excitation. Note that the time scale in the TA maps is linear up to 1 ps and logarithmic after.

TA spectra of the three samples also display pronounced spectral evolution, as evidenced by their two-dimensional TA maps (Figure 6, middle row). The negative TA features (ESA) of all samples appear to blueshift in the first picosecond, as the ESA band at short wavelengths grows while the one at longer wavelengths decays. Such a behavior is evidence of IC to an excited state below the photoexcited one, followed by vibrational cooling and structural reorganization of the polymer backbone as it accommodates and stabilizes the newly formed dark state.^29,44^ While the three samples share similar early time behavior, the most striking finding is the disparity in the transient recovery kinetics in **PICTTz** when compared to the other two samples: **PICTTz** positive GSB signal recovers almost completely within 100 ps, indicating a highly efficient, subnanosecond deactivation pathway back to the ground state. **TTz** and **PCTTz** GSB recoveries are significantly slower, persisting beyond the ∼1 ns measurement window and suggesting a lack of the deactivation pathway present in **PICTTz**. The observation of this long-lived excited state in **PCTTz** and **TTz** agrees with the results from TCSPC presented above and with previous studies.^20,22^

Global analysis as implemented in pyglotaran^45^ was used to extract the lifetimes and their corresponding spectra for the measured TA maps. To aid interpretation, both evolution-associated difference spectra (EADS) and decay-associated difference spectra (DADS) were analyzed. EADS, derived from a sequential kinetic model, provides a convenient representation of the apparent spectral evolution and population flow between states. In contrast, DADS, obtained from a parallel model, decomposes the signal into distinct kinetic components, allowing identification of processes with different time constants. The combined use of both approaches provides a consistent and complementary description of the ultrafast dynamics. The global analysis models needed four compartments to properly fit the data throughout the ∼1 ns measurement window, with the fourth compartment in all samples treated as an offset to account for long-lived signals. For **TTz**, the retrieved time constants were 0.15, 1.2, and 90 ps. For the copolymers, in **PCTTz**, the fitted time constants had values of 0.55, 16, and 35 ps, while for **PICTTz**, values of 0.22, 15, and 18 ps were retrieved. Spectra of each EADS component retrieved in the global analysis are shown in Figure 7, while the DADS is found in Figure S4. The multiexponential relaxation process in the copolymers is evidence of multiple states participating in the excited-state dynamics. The fastest EADS component for all three samples reveals a similar evolution within the first picosecond: the blue band ESA becomes more negative (evidenced in **PCTTz** as a decrease in the positive signal due to GSB/ESA overlap) while the red band ESA loses amplitude. We assign this similar early evolution as the tail-end of the IC to the dark state in all three samples, as the TRFU traces still show a fluorescence decay up to ∼2 ps. Following this early time evolution, the dynamics begin to diverge. **PCTTz** exhibits a rise of the positive signal from the second to third EADS component over the next ∼16 ps, whereas **PICTTz** shows only a decay of its positive transient signal from its second to third EADS component over ∼15 ps.

**Figure 7:**
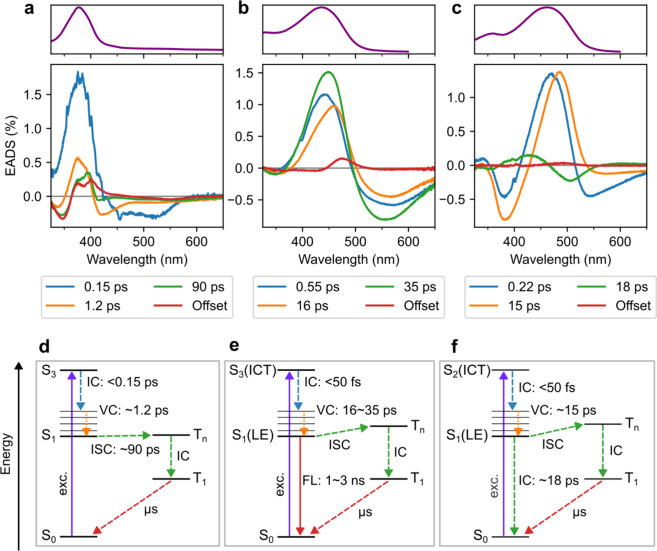
Steady-state absorption spectra (top) and fitted EADS (middle) from the TA data in Figure 6 of (column a) **TTz**, (b) **PCTTz**, and (c) **PICTTz** and their respective time constants (bottom). Relevant energy levels and assigned relaxation pathways for (d) **TTz**, (e) **PCTTz**, and (f) **PICTTz**. Solid arrows represent radiative processes and dashed nonradiative processes. The abbreviations stand for IC: internal conversion, VC: vibrational cooling, ISC: intersystem crossing, and FL: fluorescence. Subpopulation of twisted **PCTTz** segments is not shown in this scheme.

Given the possibility of exciting a subpopulation of the emissive species in **PCTTz** with the 400 nm pump, thereby potentially obscuring the pure transient response from the weakly emissive population, TA spectroscopy was also performed using a 490 nm pump. The 490 nm TA spectra (Figure S5) are similar to the 400 nm pump TA spectra after 10 ps, and global analysis yielded four EADS components with time constants of 0.13, 19.6, and 27.1 ps and an offset (Figure S6), which are broadly similar to the 400 nm excitation EADS values. Key differences emerged; however, the early time filling of the blue ESA band within the first picosecond was absent under the 490 nm pump and the later rise in the GSB signal was also absent, with its second to third EADS component showing only decay of its positive feature (Figure S6). These differences can be attributed to 490 nm excitation photoexciting conformers with a configuration closer to the relaxed excited-state configuration,^25,46^ resulting in a faster IC to the dark state as dictated by the energy gap law.^47^ The spectrum of the offset signal is nonetheless practically identical between 400 and 490 nm excitation (Figure S7), demonstrating that photoexcitation of **PCTTz** lowest absorption band always leads to population of the same long-lived state.

In the EADS for all samples, we observed a blueshift of the negative features from the second to third EADS component together with a recovery of the GSB signal in some regions. With this dynamical evolution in mind and considering its 1–16 ps time scale, we assign the second EADS component as a hot S_1_ state, which thermalizes through vibrational cooling.^44,48^ To test this hypothesis, we followed the procedure established by Kovalenko et al.^44^ and performed a selective band integration of the TA signal. Integrating over the TA signal not only increases the signal-to-noise ratio but also performing an integration of the entire GSB+ESA band yields a trace only sensitive to changes in the magnitude of the transition dipole moment (oscillator strength) and population dynamics, canceling the transients from hot populations, while integrating over a narrow band in the GSB or an ESA region also gives information about state cooling.^44,49,50^ In general, it was observed that when integrating over the GSB+ESA band of each sample and fitting the resulting trace to one to three parallel exponential decays plus an offset, the components with time constants within 10–20 ps either vanished or showed a considerable decrease in amplitude when compared to a narrow integration in the GSB region only (traces and fits in Figures S8–S10 and fitted parameters in Tables S1–S3), supporting the vibrational cooling at this time scale. Crucially, the offset signal remains present after the GSB+ESA integration in all samples, demonstrating that it cannot be assigned to a structurally hot ground state, since such a state would produce a spectrum that integrates to zero due to its opposite GSB and ESA contributions.^44^ Furthermore, the low viscosity of the solvent and long time scale of this signal (>1 ns) make it unlikely for vibrational or torsional motion to play a large role at this time scale, as they typically relax within tens of picoseconds.^34,44^

Considering the nonobserved emission from the S_1_ state in **TTz** and **PICTTz**, the long-lived (offset) signals are attributed to the formation of a triplet species due to ISC (Figure 7d).^22,23^ Since the LE S_1_ has n−π* character in all samples, enhanced spin–orbit coupling can occur to energetically close π–π* T_*n*_ (*n* > 1) states by the El-Sayed rules,^21^ which would then undergo ultrafast IC to a long-lived T_1_ and give rise to the long-lived transient (Figure 7d–f). Despite S_1_ being mostly localized in the *s*-tetrazine, the donor structure can significantly alter the efficiency of this ISC process: while in **TTz**, a large portion of the population efficiently crosses to the triplet manifold in 90 ps (evidenced by the amplitude of the offset GSB), and in **PICTTz**, only a small fraction of the S_1_ population is converted into triplets in 18 ps as an efficient S_1_→S_0_ IC competes with the ISC process (Figure 7f). Different from the previous samples, **PCTTz** exhibits a nanosecond TCSPC emission lifetime, which by its time scale must come from S_1_. Therefore, the offset signal in **PCTTz** is attributed to a mix of S_1_ and T_1_ (Figure 7e).

The measurements reported so far for the copolymers are consistent with photoexcitation to a bright ICT state followed by ultrafast IC to a dark LE S_1_ state, which then vibrationally cools in less than 20 ps (Figure 7e,f). While the transition to the ICT state is strongly allowed and dominates the steady-state absorption spectrum, this excitation is converted to *s*-tetrazine’s n−π* S_1_(LE) state in less than a hundred femtoseconds as shown in the TRFU traces, decreasing the emission yield and explaining the low FQY values reported. While most polymeric segments are sufficiently planar to permit photoexcitation to the ICT state, a subpopulation of chromophores exists in highly twisted geometries in **PCTTz**, which are preferentially excited at shorter wavelengths as shown above. These torsions electrically decouple the donor and acceptor units, disrupting the ICT state and promoting a **TTz**-like S_*n*_ emission from the copolymer, as evidenced by the increase in the FQY and the larger contribution (92%) of the 0.96 ns component in TCSPC trace when the excitation moves to shorter wavelengths. Conversely, excitation to the ICT state in **PCTTz** promotes a slower biexponential radiative relaxation, with the emission amplitude evenly split between the two components.

TA spectroscopy demonstrates significant differences in the evolution of the dark S_1_(LE) state as competition between IC and ISC takes place. While the LE state efficiently populates a triplet state in 90 ps in **TTz**, the singlet-to-triplet energy gap in the copolymers is likely unfavorable as suggested by the SOC calculations (Figure S12) and weak transient signal at long times, converting only a smaller portion of the S_1_ population into triplets through ISC. Besides the effect of the energy gap between the manifolds, the dynamics of S_1_ itself can limit the efficiency of ISC in **PICTTz**, as the transient signal almost completely recovers in tens of picoseconds due to rapid IC to the ground state depleting the S_1_ population before ISC can convert it into a triplet. This S_1_→S_0_ IC seems to depend on the structure of the donor fragment, as **PCTTz** does not exhibit such a fast IC of its S_1_ population, with the persistence of its GSB signal and the observed TCSPC emission (1–4 ns) implying that the energy level structure prevents a barrier in the excited-state potential energy surface to be crossed.^50^ Being unable to cross this barrier, **PCTTz** excited state remains in a mixture of S_1_ and T_1_, where in the former, a slower (ns) IC competes with radiative relaxation.

The mechanism of IC to a dark state shown in this work was recently proposed by Shen et al.^28^ to explain the fluorescence quenching process in tetrazine-based fluorophores. In the context of OSCs, this IC process also acts as a trap for the excitation, preventing down-chain energy transfer and negatively impacting the performance of OPV devices, as shown by Cassemiro et al. using PICTTz:PC_71_BM, which presented a low PCE of ∼1%.^31^ Other *s*-tetrazine-based OSCs reported higher PCEs in a range of 5–8% when paired with PC_71_BM,^19,32,51^ with the critical factor being the ICT state lower in energy than the LE state from *s*-tetrazine. Such a lowering in energy can be promoted by adopting synthetic strategies such as incorporating a longer thienyl chain,^28,32,51^ replacement of the donor fragment,^19^ or through quinoidization of the π-conjugated backbone,^38,52^ as the dark LE state of *s*-tetrazine has a stable transition energy of 2.3–2.4 eV under these changes.^17,28^

## 4 Conclusions

In this work we have investigated the excited-state dynamics of dithienyltetrazine (**TTz**) and two tetrazine-based donor–acceptor copolymers, **PCTTz** and **PICTTz**, combining steady-state spectroscopy, femtosecond fluorescence upconversion, transient absorption spectroscopy, and TDDFT calculations. The measurements reveal a consistent photophysical picture in which photoexcitation of the copolymers initially populates a strongly allowed π–π* transition with intramolecular charge-transfer (ICT) character that undergoes ultrafast (<100 fs) internal conversion to a tetrazine-localized n−π* singlet state. This dark S_1_ state acts as an efficient nonradiative funnel that rapidly quenches fluorescence despite the strong optical absorption of the materials.

Ultrafast spectroscopy further reveals pronounced conformational heterogeneity within the polymer chains. While the majority of conformations efficiently populate the dark tetrazine-centered state, a minor subpopulation of highly twisted segments disrupts donor–acceptor conjugation, suppressing ICT formation and enabling brighter **TTz**-like emission from higher excited states. This structural heterogeneity explains the strong excitation-wavelength dependence observed in the fluorescence spectra and highlights the critical role of backbone conformation in determining the emissive properties of tetrazine-based polymers.

Comparison between the two copolymers demonstrates that subtle variations in the donor structure strongly influence the fate of the dark singlet state. In **PCTTz**, a long-lived excited population persists beyond the nanosecond time scale, consistent with population of long-lived excited states likely involving a triplet manifold. In contrast, **PICTTz** exhibits rapid ground-state recovery within hundreds of picoseconds, indicating a highly efficient nonradiative decay pathway. These results show that donor selection can dramatically alter the relaxation landscape, even when the same tetrazine acceptor unit is present.

Together, these findings establish a mechanistic framework for understanding fluorescence quenching in tetrazine-based donor–acceptor polymers: the presence of a low-lying tetrazine n−π* state provides an ultrafast pathway that funnels excitation away from emissive π–π* states. More broadly, this work highlights how the interplay between donor–acceptor coupling, backbone conformation, and excited-state ordering governs energy dissipation in conjugated polymers. Controlling these factors will be essential for mitigating ultrafast internal conversion and for designing tetrazine-containing materials with improved performance in future organic optoelectronic and photonic applications.

## Supplementary Material



## Notes

Detailed experimental methods, TCSPC traces, TA DADS, 490 nm excitation TA data, band integration TA traces, calculated DFT geometries, TDDFT excitations, and triplets and SOCs at the S_1_ optimized geometry (PDF)
