# Supplementary material for: Ultrafast Excited-State Dynamics of Dithienyltetrazine-Based Donor–Acceptor Copolymers
Source: J Phys Chem B. 2026 May 25;130(22):5801–10. doi: 10.1021/acs.jpcb.6c01609 (PMC13244442; doi:10.1021/acs.jpcb.6c01609)
Supplement: Supplementary file 1 [file jp6c01609_si_001.pdf]

Supporting Information

Ultrafast Excited State Dynamics of Dithienyltetrazine Based Donor-Acceptor Copolymers

Erico M. Braun[1], Samylla Boazegevski[2,3], Jeferson F. de Deus[2], Cristiano Zanlorenzi[3], Sandra M. Cassemiro[3], Leni C. Akcelrud[3], Samim Sardar[4], Franco V. A. Camargo[5], Giulio Cerullo[5,6], Giovanni Bressan[7], Anam Fatima[7], Stephen R. Meech[7] and Ismael A. Heisler[1]*

[1]*Instituto de Física, Universidade Federal do Rio Grande do Sul - UFRGS, Avenida Bento Gonçalves, 9500, Porto Alegre, Brazil*

[2] *Departamento de Física, Universidade Tecnológica Federal do Paraná – UTFPR, Av. Sete de Setembro, 3165, Curitiba, Brazil*

[3]*Departamento de Química, Universidade Federal do Paraná - UFPR, 81531-990 Curitiba, Brazil*

[4]*Department of Chemistry, National Institute of Technology Warangal, Telangana 506004, India*

[5]*Istituto di Fotonica e Nanotecnologie, Consiglio Nazionale delle Ricerche, Piazza L. da Vinci 32, 20133 Milano, Italy*

[6]*Dipartimento di Fisica, Politecnico di Milano, Piazza L. da Vinci 32, 20133 Milano, Italy*

[7]*School of Chemistry, Norwich Research Park, University of East Anglia, Norwich NR4 7TJ, UK*

Corresponding author ismael.heisler@ufrgs.br

Contents

1. Detailed experimental methods……………………………………………………….S3

2. TCSPC traces of PCTTz………………………………………………………………S5

3. Copolymers TA data pump fluence dependence…………….……………………......S6

4. 400 nm excitation TA DADS………….....…..……………………………………....S7

5. PCTTz 490 nm excitation TA data and global analysis……………………………....S8

6. Band integration of TA data…………………………………………………………...S10

7. Calculated DFT ground state geometries using CPCM(Chloroform)………………S12

8. Calculated TDDFT vertical transition energies………………………………………S16

9. Comparison between absorption spectra and TDDFT excitations…………………S17

10. Optimized S_1, triplet energies and spin-orbit couplings……………………………S18

9. References……………………………………………………………………………S19

1. Detailed experimental methods

Synthesis. Synthesis goes here.

Steady-state spectroscopy. Steady-state absorption and fluorescence measurements were performed using a Lambda XLS Spectrometer (Perkin-Elmer) and a Cary Eclipse Fluorescence Spectrometer (Agilent), respectively, employing 10 mm pathlength quartz cuvettes. All samples were prepared in spectroscopy-grade chloroform (Sigma Aldrich), at concentrations adjusted to maintain an optical density (OD) of approximately 0.5 at the maximum absorption peak, ensuring minimal aggregation. For steady-state fluorescence experiments, samples were further diluted until their absorbances values were below 0.1, effectively minimizing reabsorption and inner filter effects.

Time-correlated single photon counting. Time-correlated single photon counting (TCSPC) measurements used a FS5 Spectrofluorimeter (Edinburgh Instruments). The excitation source was either the pulsed LED EPLED-365 or the pulsed laser diode EPL-485 (Edinburgh Instruments) to excite the samples at 365 or 485 nm, respectively, at a repetition rate of 20 MHz. Instrument response function (IRF) of the setup was determined to be 900 and 470 ps (FWHM) for excitation at 365 and 485 nm, respectively. Sample preparation for TCSPC was the same as for steady-state fluorescence.

Femtosecond transient absorption spectroscopy. Femtosecond transient absorption (TA) spectroscopy was carried out using a previously described experimental setup.[1,2] Briefly, a Ti:Sapphire amplified system (Libra, Coherent) producing 100 fs ultrashort pulses at 800 nm with a repetition rate of 1 kHz was used to generate both pump and probe pulses. The pump pulse was derived either from the second harmonic of the 800 nm fundamental or from the output of a custom-built optical parametric amplifier and modulated at 500 Hz by an optical chopper. For the probe, a white light continuum was generated by focusing a portion of the fundamental pulse into a continuously translated CaF_2 plate, resulting in a detection range of 325-650 nm after passing through a BG39 filter (SCHOTT).

Polarizations of the pump and probe beams were set to the magic angle (54.7°) and focused onto the sample with beam diameters of 200 µm and 100 µm, respectively. The transmitted probe after the sample was directed to a grating spectrometer (SP2150 Acton, Princeton Instruments) and detected at 1 kHz by a linear sensor camera (Stresing). The IRF of the TA system was determined by fitting the coherent artifact observed in neat chloroform,

yielding a full width at half maximum (FWHM) of 100 fs for all excitation wavelengths used. Solution samples for TA measurements were prepared in 1 mm pathlength cuvettes, with absorbance values in the range 0.3-0.5 at the excitation wavelength.

Time-resolved fluorescence upconversion. Time-resolved fluorescence upconversion (TRFU) was employed to investigate the ultrafast dynamics of the bright excited state, using a setup previously described in the literature.[3] In brief, transform-limited frequency-doubled pulses from a Ti:Sapphire oscillator (Coherent MICRA) were used to excite the sample at 400 nm. The resulting fluorescence was collected using a microscope objective imaging the cuvette, and was subsequently upconverted via sum-frequency generation in a 100 μm thick Type-I BBO crystal, using the fundamental wavelength of the oscillator as the gating pulse. Upconverted photons were detected at a specific wavelength using a photomultiplier tube coupled to a monochromator. The instrument response function (IRF) of this setup exhibited a full width at half maximum (FWHM) of 82 fs. Sample preparation for TRFU measurements was identical to that used for TA.

2. TCSPC traces of PCTTz

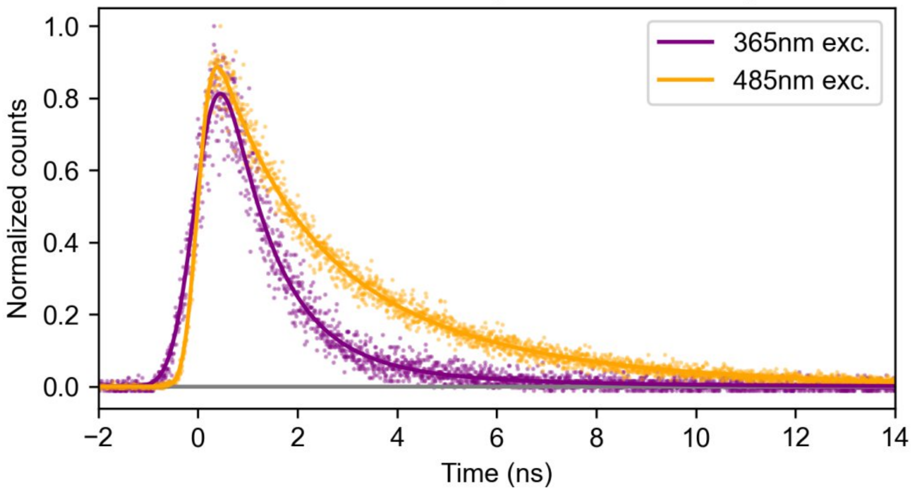

Figure S1. PCTTz TCSPC emission under 485 and 365 nm excitation and 535 nm detection. Fit (solid lines) to a biexponential with time constants (amplitudes): 0.96 ± 0.02 (92%) and 3.1 ± 0.3 ns (8%) for 365 nm excitation (purple); 1.42 ± 0.02 (51%) and 3.79 ± 0.05 ns (49%) for 485 nm excitation (orange).

3. Copolymers TA data pump fluence dependence

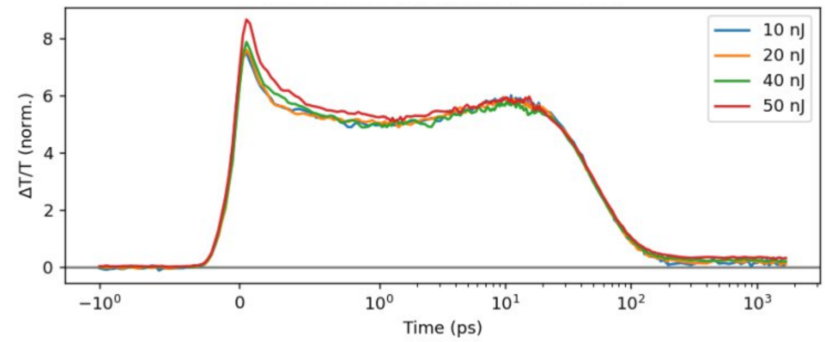

Figure S2. PCTTz GSB transient signal under 10, 20, 40 and 50 nJ pump energies, corresponding to 8, 16, 32 and 40 μJ/cm² fluence, respectively.

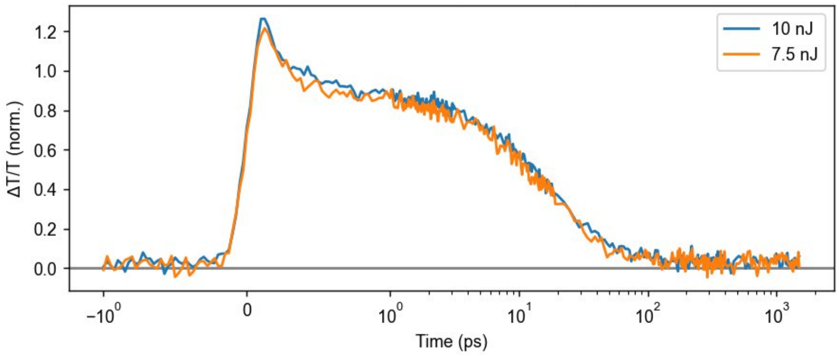

Figure S3. PICTTz GSB transient signal under 7.5 and 10 nJ pump energies, corresponding to 6 and 8 μJ/cm² fluence, respectively.

4. 400 nm excitation TA DADS

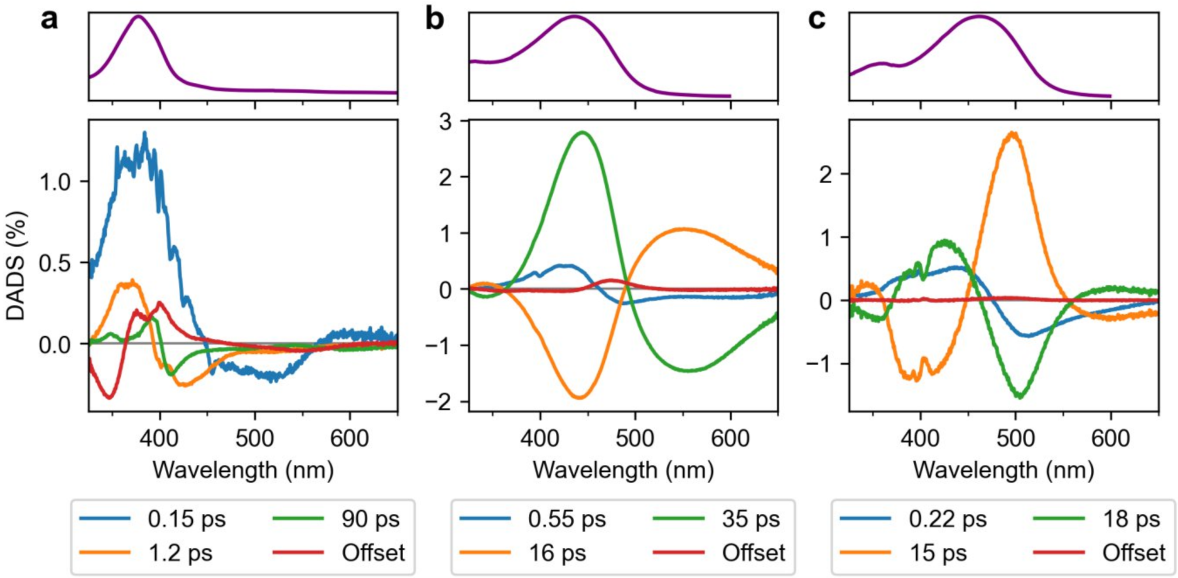

Figure S4. Steady-state absorption spectra and DADS from global analysis of 400 nm excitation TA data of **TTz** (a), **PCTTz** (b) and **PICTTz** (c).

5. PCTTz 490 nm excitation TA data and global analysis

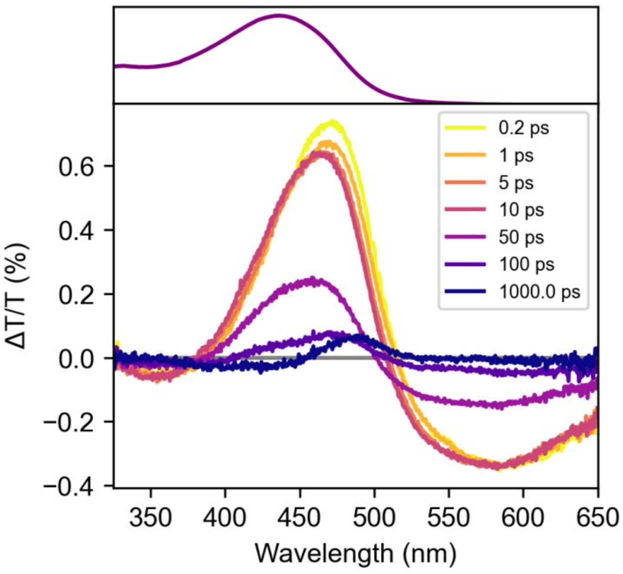

Figure S5. TA spectra for **PCTTz** under 490 nm excitation at selected delay times.

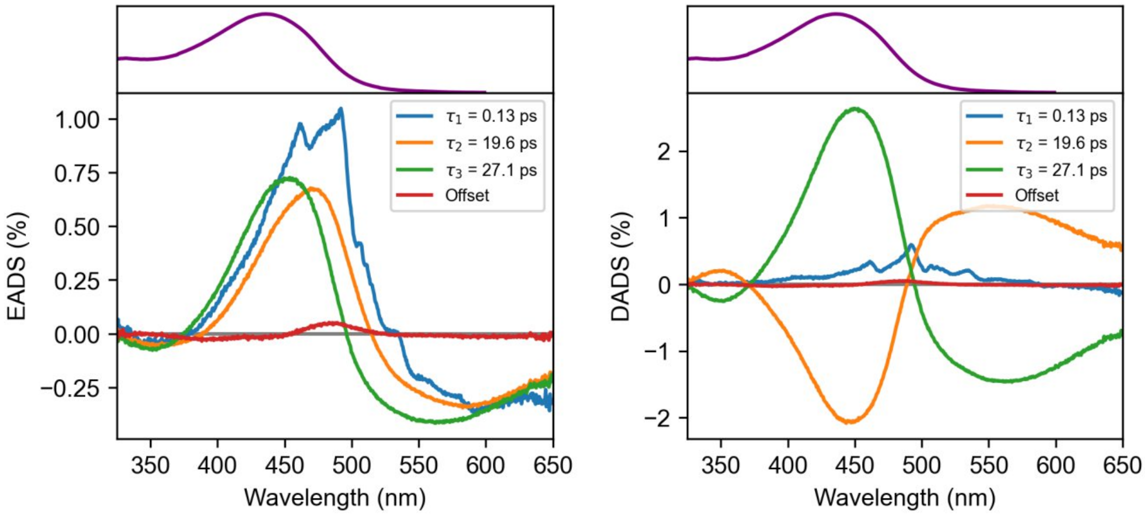

Figure S6. EADS (left) and DADS (right) for **PCTTz** under 490 nm excitation.

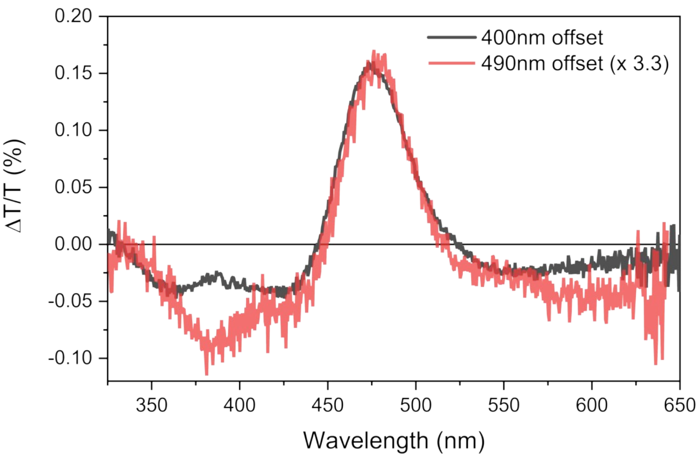

Figure S7. Comparison between the offset signals in 400 and 490 nm excitation TA data.

6. Band integration of copolymers TA data

Band integration of TA data followed the expression defined in Kovalenko et al.[4]

$$\mathrm{BI}\,(\lambda_1,\lambda_2) = \int_{\lambda_1}^{\lambda_2} \frac{\Delta T}{T}(\lambda,t)\,\frac{d\lambda}{\lambda},$$

with the integration interval (λ_1,λ_2) covering either the GSB+ESA region of the samples (tracking population decay and oscillator strength changes) or only a narrow band in the GSB region (cooling dynamics). The choice for the GSB+ESA wavelength range was to cover the gaussian-derivative shape in the second DADS component. In **TTz**, the band integrated traces were fitted to either an autocorrelation term, one exponential decay and an offset or two decays and an offset. In **PCTTz** and **PICTTz**, the band integrated traces were fitted to the sum of a gaussian autocorrelation function, two to three exponential decays and an offset.

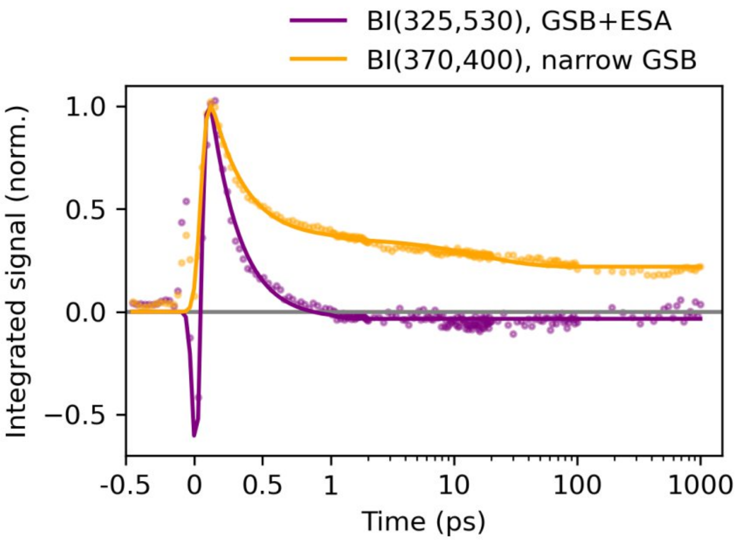

Figure S8. Band integrated TA traces of **TTz** under 350 nm excitation. Fit: solid lines.

Table S1. Fitted parameters in **TTz** band integrated TA trace. Amplitudes relative to signal peak.

Trace	A_{corr} (%)	A_1 (%)	τ_1 (ps)	A_2 (%)	τ_2 (ps)	A_{offset} (%)
BI(325,500)	-264	167	0.22	-	-	-3.6
BI(370,400)	-	71.5	0.25	11.3	17	9.4

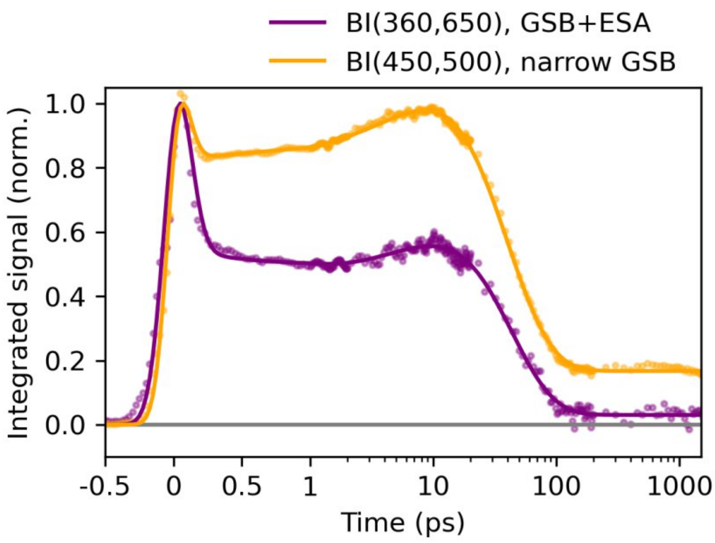

Figure S9. Band integrated TA traces of **PCTTz** under 400 nm excitation. Fit: solid lines.

Table S2. Fitted parameters in **PCTTz** band integrated TA trace. Amplitudes relative to signal peak.

Trace	A_{corr} (%)	A_1 (%)	τ_1 (ps)	A_2 (%)	τ_2 (ps)	A_3 (%)	τ_3 (ps)	A_{offset} (%)
BI(360,650)	71.6	4.9	0.7	-35.2	11	57.0	31	1.6
BI(450,500)	53.4	-	-	-48.8	9	85.9	31	9.4

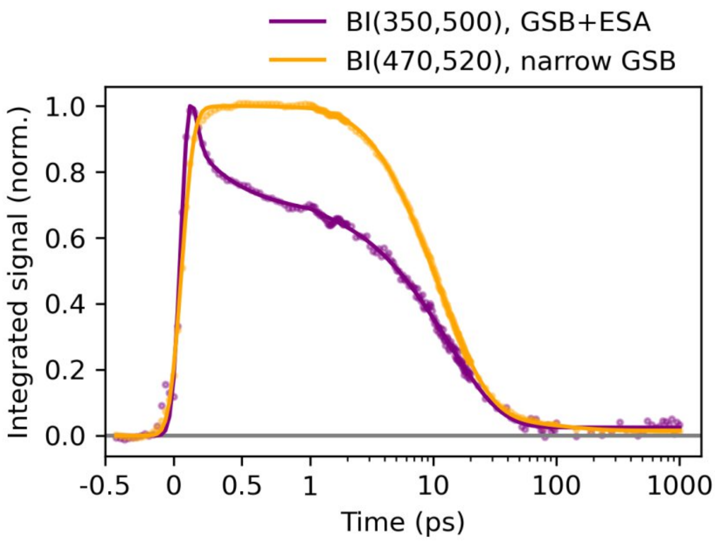

Figure S10. Band integrated TA traces of **PICTTz** under 400 nm excitation. Fit: solid lines.

Table S3. Fitted multiexponential parameters in **PICTTz** band integrated TA trace. Amplitudes relative to signal peak.

Trace	A_{corr} (%)	A_1 (%)	τ_1 (ps)	A_2 (%)	τ_2 (ps)	A_3 (%)	τ_3 (ps)	A_{offset} (%)
BI(350,500)	47.9	11.4	0.3	39.3	13	-	-	1.4
BI(450,500)	53.4	-22.2	1.6	115.6	10	5.0	85	1.5

7. Calculated DFT ground state geometries using CPCM(Chloroform)

TTz

C	-2.03656714746234	0.44126427746505	-0.00001404634803
N	-1.16080563259179	1.45417742237927	-0.00020238740205
N	0.10545436874798	1.16080313809757	-0.00024601252668
N	-1.68226084330040	-0.85019615499544	0.00008569483164
N	-0.41577199864610	-1.14397395047485	0.00003653655123
C	0.46012684258662	-0.13111777489348	-0.00011177549058
C	-3.46053491821957	0.75322338725977	0.00007254338025
C	1.88344272833512	-0.44252711913470	-0.00011058681125
S	3.05393847885764	0.81828643099003	-0.00013812861408
C	4.31706851554353	-0.33330031622879	-0.00014159970347
C	2.46848906843857	-1.67933933645483	-0.00005025841204
C	3.89261987546527	-1.63321818750479	-0.00008759790617
S	-4.63237697452257	-0.50758631071539	0.00037010357902
C	-5.89703651496974	0.64695180259520	0.00025692851894
C	-5.47104606963202	1.94518794807057	0.00007555778690
C	-4.04374669587956	1.98827805315073	-0.00002977572477
H	-3.47444496475741	2.90904989227161	-0.00019895657024
H	1.90028234035690	-2.60060569890355	-0.00002501010767
C	-6.34062819034477	3.16516145041873	-0.00004868749462
H	-7.39700013863967	2.89780798326779	0.00024759561653
H	-6.13807578047961	3.77655234102289	-0.88287906372538
H	-6.13769176882564	3.77700759040679	0.88237621945334
Br	-7.66421932866287	0.04223625454308	0.00043174732757
Br	6.09280128984070	0.23725338941424	-0.00009142281349
C	4.79274322949463	-2.83221683811784	0.00005000708961
H	4.20485731591285	-3.75048807279753	-0.00208476749076
H	5.43702589981070	-2.83399372254578	0.88281070024777
H	5.44020701354352	-2.83184787858633	-0.88036355724149

PCTTz

C	-7.41688023263751	1.36411747046345	-0.23192278470894
C	-6.93322404867456	2.50452519278987	-0.88962420101325
C	-5.57220335466709	2.68326096112345	-1.08168229700959
C	-4.69118912813958	1.70972767945848	-0.61225387960940
C	-5.19327564573338	0.56288579085009	0.04135688340679
C	-6.56180980921521	0.38128821477556	0.24308202504078
C	-3.24631344385293	1.57238032720728	-0.62724697773525
N	-4.13643130970122	-0.25753224527104	0.40053042818060
C	-2.95440397861750	0.35005686020663	0.01710712791830

C	-2.20338950306209	2.35709125760680	-1.11480105224399
C	-0.89827734962015	1.92593151394189	-0.94836354903068
C	-0.61202504247973	0.71459931737996	-0.29040909526878
C	-1.64390354019554	-0.08463330392493	0.19717222291154
H	-5.19603197453639	3.56691479948648	-1.58695509653206
H	-8.48593546776390	1.24769199008392	-0.08818474886878
H	-7.63284385189918	3.25205079445141	-1.24670921289799
H	-6.94957686859056	-0.49139355564402	0.75543534891654
H	-2.40903218035575	3.29278686563700	-1.62410927984129
H	-0.08166594933320	2.51748866082352	-1.34341921771181
H	-1.42126631376826	-1.00811276852924	0.71864745596985
C	-4.24395938766770	-1.49299689792499	1.14498720461111
H	-5.17977632032936	-1.99044395236331	0.89018627436607
H	-4.21587191335933	-1.31584189011175	2.22446619366431
H	-3.42202431324855	-2.15487363621869	0.87174347474765
C	0.78865933387694	0.27787801225817	-0.13272102583210
C	1.88600344253066	1.01535891491109	0.24849921680440
C	3.06405787499468	0.21749722094199	0.28541685089647
C	2.84580896531705	-1.08737514644279	-0.06316826053444
S	1.19486190348947	-1.36413123799985	-0.44528009710225
H	4.03693407983957	0.59515624318615	0.57377322331475
C	1.87737707206814	2.47067257815139	0.62430799166787
H	0.90351185075154	2.77455573493011	1.01037001081335
H	2.10749950884337	3.10332159829193	-0.23775354908629
H	2.63195595394727	2.66494902564055	1.38916812250811
C	3.82278455460970	-2.16486102487481	-0.12621121307159
N	5.10138734814249	-1.88569290394677	0.16268039748686
N	3.38991612188396	-3.38503154272850	-0.47248506421720
N	4.25431202747123	-4.35445428844217	-0.53252725421975
N	5.96453749858582	-2.85585496101836	0.10440785772260
C	5.53150063262875	-4.07557082732918	-0.24179401090850
C	6.50965876338883	-5.15523073442077	-0.30381453646469
S	8.16619814351243	-4.86980329359950	0.05682438775344
C	6.28449566693208	-6.46403686157993	-0.63201784414071
C	7.46847104615725	-7.25813416149941	-0.59378065621847
C	8.55314830469041	-6.50812880969282	-0.23390860818551
H	5.30546214540250	-6.84619381883532	-0.89198652802426
H	9.57414371900501	-6.84692811108948	-0.12524277392186
C	7.50418644841724	-8.72582852035464	-0.90956887990780
H	6.85556610587821	-9.28441524942238	-0.22956011776347
H	8.51774217332461	-9.11914045259701	-0.81854954096768
H	7.15249024175938	-8.91174682873536	-1.92781134566287

PICTTz

C	-7.29115935072515	1.82290882624031	-0.19233306356047
C	-6.73701994759982	2.94207777069413	-0.87032277232761
C	-5.37111177310726	3.08214363406642	-1.07492521342897
C	-4.56051874926492	2.05669856389259	-0.59368658561241
C	-5.11594478612297	0.92979208465297	0.06906886229259
C	-6.48100065907180	0.79689256818166	0.28656622730491
C	-3.12357587815261	1.85073240528297	-0.60647664638407
N	-4.08735416359669	0.06345605203150	0.42851763713493
C	-2.88372293930155	0.61816724367304	0.04288194871141
C	-2.05068703719386	2.58916197926158	-1.09949512476604
C	-0.76306062913108	2.10652509939814	-0.93534626669821
C	-0.52701210393733	0.88730492428842	-0.27302073195151
C	-1.59008417787613	0.13318417663451	0.22057551079074
H	-4.94824137793191	3.94500951535665	-1.57662965790059
H	-6.90180300777188	-0.05543177990169	0.80767503196904
H	-2.21985842698289	3.53054311753529	-1.61203541519728
H	0.07596671996213	2.66235264986457	-1.33519445442771
H	-1.40176139830211	-0.79577737357700	0.74570756012042
C	-4.26368279652546	-1.15918460314223	1.17814610835219
H	-5.15467743368195	-1.68048394754468	0.82422768381826
H	-4.36884728584153	-0.96341574766820	2.25006418820806
H	-3.40472721080384	-1.81013420366840	1.01907443951709
C	0.85441438539052	0.39254893147157	-0.11712163620723
C	1.98368839406680	1.08482329152204	0.25604541982514
C	3.12704580715915	0.23809706928658	0.29261515091920
C	2.85256505966612	-1.05821751465459	-0.04827988213015
S	1.18989814447469	-1.26682586752363	-0.42239414665527
H	4.11617761927555	0.57571334348077	0.57533701640877
C	2.03859599712548	2.54103367764767	0.62448707998488
H	1.08055501938277	2.88764126170334	1.01409669266251
H	2.29044505506749	3.15930832408944	-0.24192250018758
H	2.80492152254435	2.70689766246027	1.38435605641505
C	3.78283287386512	-2.17629895122855	-0.10979593120107
N	5.07354752932868	-1.95004560600492	0.17167589061642
N	3.29724315187378	-3.37890688600129	-0.44776612292770
N	4.11963375626993	-4.38425177103353	-0.50689682219661
N	5.89463728124960	-2.95611683017163	0.11429812393843
C	5.40887029017649	-4.15815595626596	-0.22359880525154
C	6.34028404483949	-5.27851812828252	-0.28469705003077
S	8.00969548011793	-5.06076644405383	0.06302387879683
C	6.05807697624937	-6.57878192852242	-0.60247659503557
C	7.20774503432504	-7.42190025546660	-0.56631439689659
C	8.32549213253879	-6.71597843784748	-0.21856157935108
H	5.06202079978510	-6.92092501234104	-0.85337596930209

H	9.33200100789805	-7.09673697395493	-0.11381554054492
C	7.17879485499033	-8.89172902470677	-0.87268655373944
H	6.50511641665905	-9.41650490950690	-0.19014901811175
H	8.17368170852013	-9.32933116497804	-0.77783868456735
H	6.82038811435702	-9.06840211511665	-1.89026405626293
N	-7.76696750212947	3.79739046703084	-1.24781290340162
C	-8.96645927569934	3.27575810702861	-0.80112342226999
C	-8.72653369158124	2.04428472830050	-0.15037516722822
C	-10.25847451832486	3.79096304543376	-0.91812549846614
C	-11.30082715281879	3.04799026337582	-0.38394641280126
C	-11.07732908554346	1.82142648390507	0.25705250478558
C	-9.79148040027181	1.31634423232311	0.37692703788898
H	-10.44973488984761	4.74031423607475	-1.40446141703873
H	-12.31371366518648	3.42891514748605	-0.46316557015425
H	-11.91711548360828	1.26865696270649	0.66320891471719
H	-9.61433242124301	0.36900537983526	0.87617121256424
C	-7.57806708352486	5.07569068824894	-1.89357029090558
H	-6.81344399268761	4.98781847608342	-2.66764998612700
H	-7.27241702776822	5.84723132471249	-1.17936423019504
H	-8.50847785400110	5.38265771790197	-2.36951405630053

8. Calculated TDDFT vertical transition energies

Table S4. The ten lowest vertical singlet transitions in **TTz**.

Transition	Energy (eV)	Wavelength (nm)	Oscillator strength *f*
1	2.59427	477.9	0.011150797
2	3.73506	331.9	0.000011345
3	3.84818	322.2	1.551428444
4	3.94934	313.9	0.071874473
5	4.79375	258.6	0.000806361
6	4.9621	249.9	0.000114067
7	5.15306	240.6	0.350901205
8	5.19126	238.8	0.000409116
9	5.20825	238.1	0.000000746
10	5.51338	224.9	0.074902725

Table S5. The ten lowest vertical singlet transitions in **PCTTz**.

Transition	Energy (eV)	Wavelength (nm)	Oscillator strength *f*
1	2.59534	477.7	0.009424481
2	3.7528	330.4	0.007993544
3	3.79791	326.5	1.915622454
4	3.90824	317.2	0.025998995
5	4.34302	285.5	0.086655935
6	4.61609	268.6	0.166005855
7	4.88453	253.8	0.029784193
8	4.94531	250.7	0.019867615
9	5.11941	242.2	0.229919182
10	5.2493	236.2	0.082065367

Table S6. The ten lowest vertical singlet transitions in **PICTTz**.

Transition	Energy (eV)	Wavelength (nm)	Oscillator strength *f*
1	2.59512	477.8	0.009398801
2	3.73623	331.8	2.186113192
3	3.75373	330.3	0.067728172
4	3.80949	325.5	0.356001064
5	3.90071	317.9	0.018276835
6	4.28048	289.7	0.349650480
7	4.63222	267.7	0.002505586
8	4.82438	257.0	0.037527641
9	4.89067	253.5	0.000730788
10	5.06229	244.9	0.085768167

9. Comparison between absorption spectra and TDDFT excitations

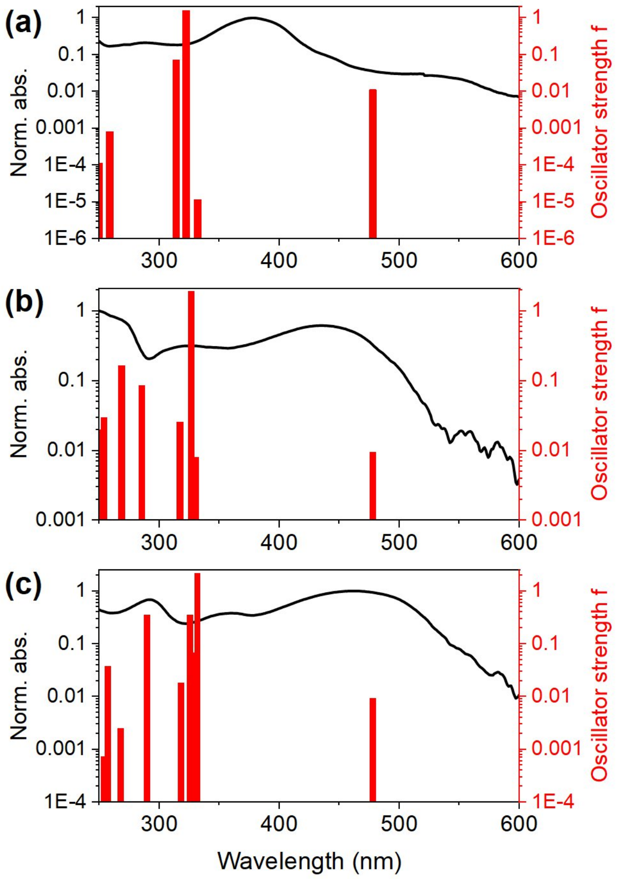

Figure S11. Comparison between the steady-state absorption spectra (log scale) with the calculated oscillator strengths (log scale) by TDDFT from the optimized ground state geometry for **(a) TTz**, **(b) PCTTz** and **(c) PICTTz**. The bright $f > 1$transitions appear relatively higher in energy than the absorption peaks in the copolymers, which is attributed to the calculations considering only one donor-acceptor pair.

10. Optimized S_1, triplet energies and spin-orbit couplings

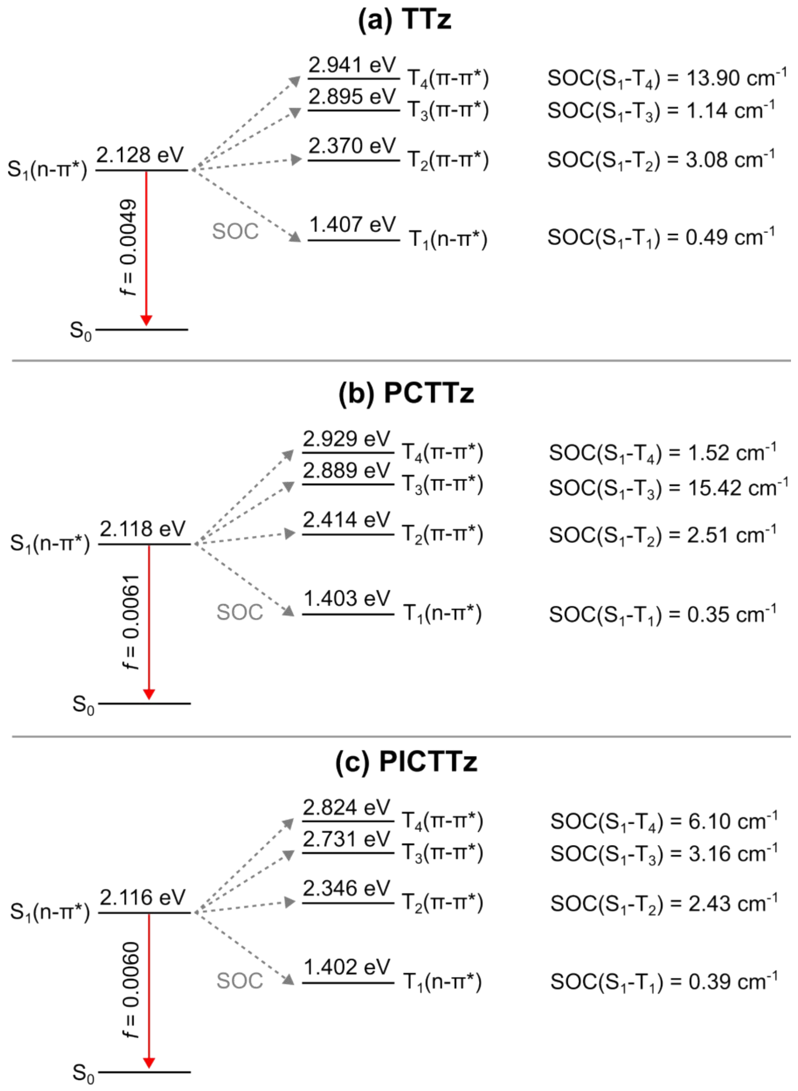

Figure S12. Calculated S_1 excitation energy, $S_1 \rightarrow S_0$ oscillator strength (*f*), triplet energies and spin-orbit coupling (SOC) at the S_1-optimized geometry at the ωB97X-D3/def2-TZVP level of theory for **TTz (a)**, **PCTTz (b)** and **PICTTz (c)**.

11. References

(1) Cerullo, G.; Manzoni, C.; Lüer, L.; Polli, D. Time-Resolved Methods in Biophysics. 4. Broadband Pump—Probe Spectroscopy System with Sub-20 Fs Temporal Resolution for the Study of Energy Transfer Processes in Photosynthesis. *Photochem. Photobiol. Sci.* **2007**, *6* (2), 135–144. https://doi.org/10.1039/b606949e.

(2) Polli, D.; Lüer, L.; Cerullo, G. High-Time-Resolution Pump-Probe System with Broadband Detection for the Study of Time-Domain Vibrational Dynamics. *Rev. Sci. Instrum.* **2007**, *78* (10), 103108. https://doi.org/10.1063/1.2800778.

(3) Heisler, I. A.; Kondo, M.; Meech, S. R. Reactive Dynamics in Confined Liquids: Ultrafast Torsional Dynamics of Auramine O in Nanoconfined Water in Aerosol OT Reverse Micelles. *J. Phys. Chem. B* **2009**, *113* (6), 1623–1631. https://doi.org/10.1021/jp808989f.

(4) Kovalenko, S. A.; Schanz, R.; Hennig, H.; Ernsting, N. P. Cooling Dynamics of an Optically Excited Molecular Probe in Solution from Femtosecond Broadband Transient Absorption Spectroscopy. *J. Chem. Phys.* **2001**, *115* (7), 3256–3273. https://doi.org/10.1063/1.1380696.
